# Otoprotection in guinea pigs exposed to pesticides and ginkgo biloba

**DOI:** 10.1590/S1808-86942012000300020

**Published:** 2015-10-14

**Authors:** Andréa Dulor Finkler, Aron Ferreira da Silveira, Gisiane Munaro, Crisley Dossin Zanrosso

**Affiliations:** a Speech and Hearing Therapist; Specialist in Clinical and Occupational Audiology (CEFAC); MSc in Human Communication Disorders (UFSM). (Speech and hearing therapist at Concordia Special Teaching Unit and Adjunct Professor – Speech and Hearing Program - ULBRA).; b PhD (Full Professor – Department of Morphology/Health Sciences Center - UFSM).; c Speech and Hearing Therapist; MSc in Human Communication Disorders - UFSM. (Medical Student - ULBRA).; d Medical Student - ULBRA).

**Keywords:** cytoprotection, toxicity, ginkgo biloba, organophosphorus compounds

## Abstract

Pesticides are widely used in agriculture, despite the risk of hearing loss related to the exposure to their chemical components. This study looks into protective drugs to counteract the ototoxicity of pesticides.

**Objective:**

This study aims to analyze the effect ginkgo biloba extract may have in protecting against possible cochlear damage caused by organophosphate pesticides (methamidophos). Anatomic changes are assessed through surface and electron microscopy.

**Materials and Methods:**

This is a prospective experimental study. Twenty-one guinea pigs were given saline solution, pesticide, and ginkgo biloba alone or combined for seven consecutive days. Then their cochleas were removed and examined in a scanning electron microscope.

**Results:**

Pesticide-exposed guinea pigs had morphological alterations in their cochleas and injuries in the three turns analyzed through electron microscopy. Injury intensity varied according to the dosages of the agents given to the test subjects. Guinea pigs treated with pesticide and ginkgo biloba maintained the architecture of their outer hair cells in all cochlear turns.

**Conclusion:**

The antioxidant properties found in the ginkgo biloba extract protected guinea pigs from pesticide ototoxicity.

## INTRODUCTION

The agricultural work in Brazil is a significant and profitable activity in social terms as well as in the business field. However, the worker's healthy in this sector requires attention due to the exposition to noise, vibration and chemical products, which are potentially harmful for the inner year^1^.

Approximately three million human are poisoned by pesticides in the whole world per year, leading to around 350.000 human deaths and two thirds of the deaths occur in developing countries^2^. In Brazil, the occurrence of poisoning by pesticides from the organophosphate group continues very high, although there has been a reduction in its use in relation to the eighty years. In the present time, about 2,5 to 3 million tons of pesticide are annually used in agriculture worldwide^3^.

The innumerable alterations provoked through poisoning caused by the organophosphate in the human being are well known. Although there is agreement in the literature regarding the existing association between the exposition to the organophosphate and auditory alterations, the studies are scarce to evaluate the use of drugs known as otoprotectors in the histological alterations in the auditory system. Several drugs have been tested to promote otoprotection as follows: sodium thiosulfate, dietilcarbamato, ACTH and derivatives, 4-metilthiobenzoic acid, lipoic acid, glutathione and its esters, methionine, procaine, alpha-melanocyte stimulating hormone (melatonin), antioxidants such as ginkgo biloba and fosfomycin and sulfur compounds^4^, ^5^, ^6^. The anatomical-physiological similarity between the peripheral auditory system of human beings and guinea pigs has encouraged researches utilizing these animals, once the results can be correlated to the humans and thus, serve as an alert for the possible injury caused by the contact with organophosphate.

According to what was presented above, the use of guinea-pig is justifiable with the objective in this research of verifying the otoprotector effect of drugs that have already been largely used in the clinical practice with other purposes, as the Ginkgo biloba extract (EGB 761) is. Concerning the cochlear damages caused by the organophosphate – methamidophos, using harmful doses to the outer hair cells, we evaluated the anatomical changes through the SEM (scanning electron microscopy) comparing the cochleae in animals under the Ginkgo biloba effects and animals without it.

## MATERIALS AND METHODS

### Study Outline

Experimental study carried out with 21 guinea pigs (*Cavia porcellus*), developed at Faculdade de Medicina de Ribeirão Preto da Universidade de São Paulo (FMRP–USP), totalizing 42 cochleae.

The guidelines from the Institute of Laboratory Animal Resources for use of Animals in the Laboratory were followed by the Brazilian College of Experimentation with Animals (COBEA), and also ethical principles of biosafety. The research project was firstly submitted under analysis and approval of Animal Experimentation Ethics Committee (CETEA) from FMRP-USP, approved under protocol number 175/2009.

The animals were selected from The Central Biotery at FMRP-USP. The inclusion criteria for the guinea pigs in the sample was the presence of the Preyer's Reflex (ear pinna contraction upon a sound stimulus) and distortion product otoacoustic emissions (DPOAE) and weight between 400 and 600 gramas. After 24 hours of auditory resting, the animals were evaluated through otoscopy of the external acoustic meatus. Those who presented otitis or cerume or an external meatus too tight to accommodate the ear probe were released. Those who had present DPOAE were selected and those who didn't were excluded.

The drugs used in the study were:

• Ketamina (65 mg/Kg).;
• Saline solution at 0.9%;
• Tiopental (Thionembutal^®^);
• Methamidophos;
• Ginkgo biloba extract

The guinea pigs were divided into five groups:

**Group 1 (Control Group) -** made up by 3 lab animals (6 cochlea), to which a 0.9% saline solution was intraperitoneally injected during 7 consecutive days;
**Group 2 (Control Group) -** made up by 2 lab animals (4 cochlea), to which a 0.3 mg/kg/day of methamidophos was intraperitoneally injected during 7 consecutive days;
**Group 3 (Study Group) -** made up by 7 lab animals (14 cochlea), to which a Ginkgo biloba extract 100 mg/kg/day was orally administered and, 90 minutes latter, methamidophos 0.3 mg/kg/day was intraperitoneally injected during 7 consecutive days;
**Group 4 (Study Group) -** made up by 2 lab animals (4 cochlea), to which was administered methamidophos 3,0 mg/kg/day intraperitoneally during 7 consecutive days;
**Group 5 (Study Group) -** made up by 7 lab animals (14 cochlea), to which a Ginkgo biloba extract 100 mg/kg/day was orally administered and, 90 minutes latter, was administered methamidophos 3,0 mg/ kg/day intraperitoneally, during 7 consecutive days;

The organophosphate methamidophos chosen is commercialized by Fersol^®^ Ltda, being the mostly utilized drug at agriculture (90%) in rural community of Córrego de São Lourenço^7^. Symptoms of intoxication referred by the labors were itching, facial flush and eyes and pharyngeal mucosal irritation.

In order to evaluate the otoprotection in the outer hair cells (OHC) exposed to lesions through methamidophos, it was used the Ginkgo biloba extract (EGb761), commercialized in Brazil as TEBONIN^®^ – Byk Química, 40mg/ml. This has been largely used in clinical practice and there are no important collateral effects, and interactions among other drugs has been well described^8^.

The minimal methamidophos dose used to be ototoxic was the same used in a previous study^9^ and confirmed through our control group. Ginkgo biloba was able to cause otoprotection to OHC in previous exposure to a potential ototoxic drug^8^, ^10^ and it was based on this studies that we used the same dose.

The lab animals were daily weighed immediately before the drug administration. To inject the drugs, it was used disposable insulin syringe BD^®^, size 21G1 (25 x 8 – 0.8 x 25 mm).

### Morphological Evaluation

In the following day after the administration of the drugs in each group, they were killed through a Tiopental injection (Thionembutal^®^) intraperitoneally, and slaughtered with the cochleae removed immediately and opened, exposing its content.

Next, the cochleae were perfused with a fixation solution of 2.5% glutaraldehyde through the round window in order to preserve the vestibulochoclear structures. After that, the material was washed five times with phosphate buffer solution 0,1M and it was then mycrodissecated in order to expose the cochlear turns. It was again emerged in a phosphate buffer solution 0,1M for 12 hours and after washed with the same solution. It was then reaffixed in a solution constituted of osmium tetroxide in 1% phosphate buffer solution 0,1M during 1 hour at 4°C. Following, it was washed in 3 baths of bidestilated water for 2 to 3 minutes each and immersed in aqueous tannic acid solution at 1% per 1 hour also at 4°C.

The pieces were all dehidratated with successive baths of ethanol in crescent concentrations of 50%, 70%, 90% e 95% during 10 minutes each. After, it was used ethanol at 100% in 3 successive baths of 20 minutes each, leaving the pieces at environment temperature for 12 hours in the last bath.

After dehidratation, the remaining water at the pieces was dry through the equipment BAL-TEC – CPD 030 – Critical Point Dryer (Balzers, 40 Liechtenstein), through the critical point process, and the pieces were removed to the chamber of the equipment and covered by liquid carbon dioxide (CO_2_).

To be better visualized at SEM, the cochleae were fixed by its bottom in a cylindrical metal with conductive carbonic paste, Colloidal Graphite (Electron Microscopy Sciences – Hatfield, PA). The structures to be analyzed were covered by a thin lack of 24 kilates gold, through evaporation process using the equipment BAL-TEC – SCD 050 – Sputter Coater (Balzers, Liechtenstein), making it electrically conductive, following studies that had showed it before^8^, ^9^, ^11^, ^12^. After all this preparation process, the pieces were then taken to be analyzed at SEM by the equipment JEOL Scanning Electron Microscope – JSM 5200 (Tokyo, Japan).

The structural analysis was standardized by the study of the medial third of the first three cochlear turns. The apical turn was not analyzed because its natural disarrangement, which could obscure the results^12^. Once the SEM was applied at the Corti's organ, the image taken was frozen and photographed to be later analyzed carefully, in respect to the OHC integrity or lesion.

The OHC integrity was defined by the stereocilia analysis. Cells presenting stereocilia well-shaped and in a normal arrangement were considered as been in integrity (normal), while those with absent stereocilia, bad shaped, deformed or in a disarrangement pattern were considered injured^8^, ^13^.

In concerned to the DPOAE, it was considered its presence or absence. It was adopted the randomized experimental design with a different number of repetitions (2 to 3 guinea pigs to the control group and 7 guinea pigs to each one of the groups exposed to the methamidophos and to the EGb).

## RESULTS

In this section, the results are presented about the otoprotector effect of the Ginkgo biloba extract in the guinea pigs submitted to the acute exposure of the ototoxic methamidophos in the periphery auditory system of these lab animals. The results of SEM, after photographed, were analyzed by observing the number of outer hair cells of the cochlea's basal turn and its arrangement pattern.

**Figure 1:**
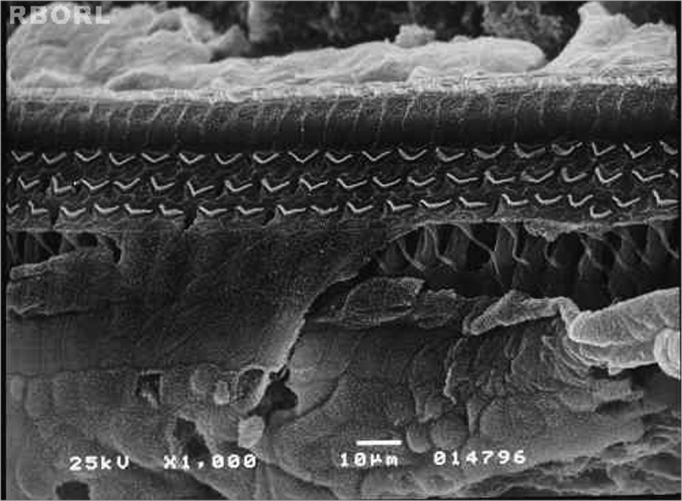
Photomicrography of a normal Corti's organ found in the guinea pig of group 1, showing its basal turn. Observe the uninjured structures in A: digital extension of the Deiter's cell; B: outer hair cells; C: outer hair cells estereocilia; D: reticular membrane. Magnified 1000x.

The damaged induced by the pesticide in the periphery auditory system was identified through the SEM of the cochleae. In the group 1 (control group – saline solution 0,9%), it was observed the maintenance of the normal OHC architecture (Figure 1).

Analyzing the groups which the pesticide were administered, the SEM evidenced morphological alterations in 100% of the cochleae in group 2 (0,3 mg/ Kg/dia) and group 4 (3 mg/Kg/dia), being the more extensive lesions in the last group.

**Figure 2:**
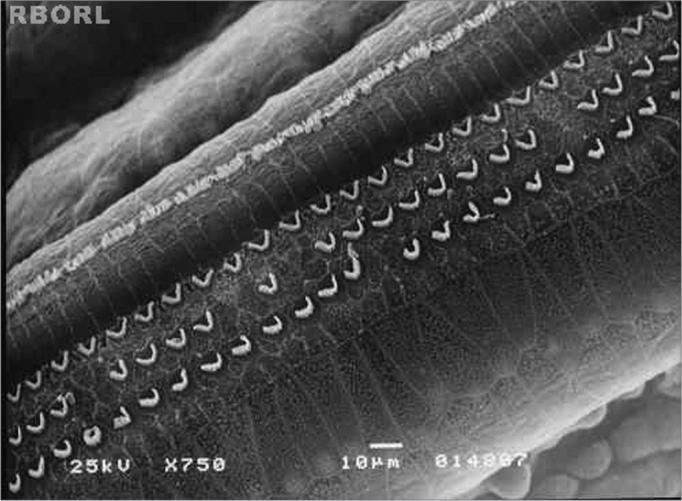
Photomicrography of the Corti's organ in a guinea pig of group 2, showing the E2 turn. Observe the outer hair cells (CCE) absence in F2 and F3. CCI: inner hairy cells; CCE: outer hair cells; F1: turn 1; F2: turn 2; F3: turn 3. Magnified 750x.

In group 2 (control group - 0,3 mg/Kg/day of intraperitoneal methamidophos during 7 days), the anatomical changes found more frequently were the OHC lesion in turns E2 and E3, evidenced by cilia distortion, derangement of the W pattern and shortening of the cilia or yet, its absence. These alterations were observed in turns 1, 2 and 3. Figure 2 shows it.

**Figure 3:**
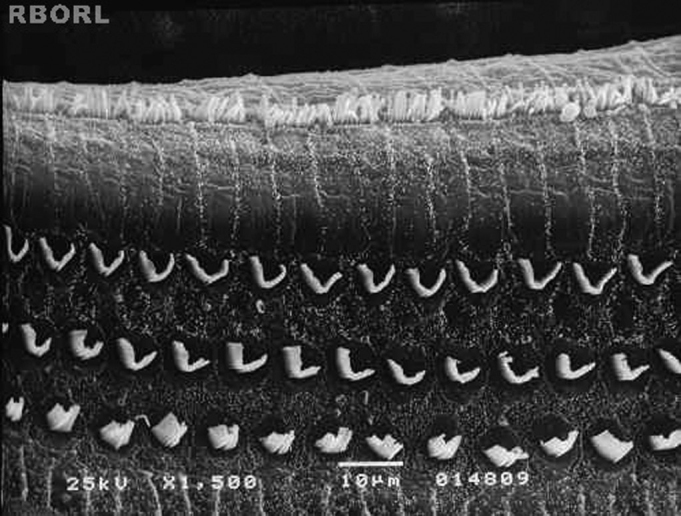
Photomicrography of the Corti's organ in the guinea pig of group 3, showing the E2 turn. Observe the normal arrangement of the outer hair cells. Magnified 1500x.

In group 3 (Ginkgo biloba 100mg/kg orally administered 90 minutes before methamidophos 0,3 mg/Kg/day during 7 days) there were no significant damage in the OHC when observed through SEM in any of the studied cochleae (Figure 3).

**Figure 4:**
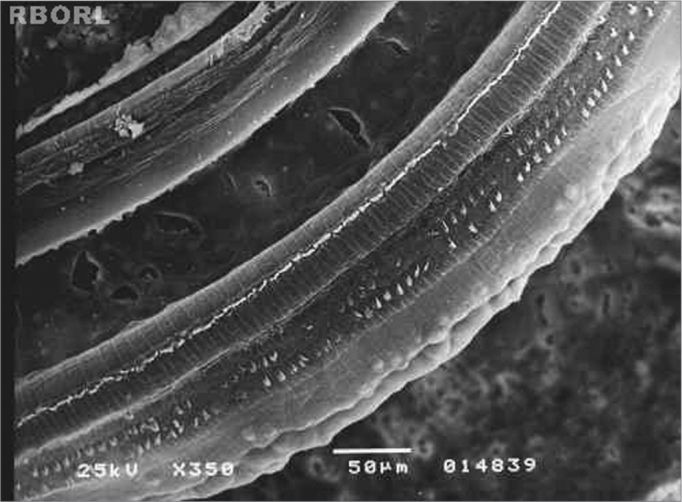
Photomicrography of the Corti's organ in a guinea pig of group 4, showing the E3 panoramically. Observe the outer hair cells absence in F1, F2 and F3. Magnified 350x.

The morphological alterations found in group 4, which received methamidophos 3,0 mg/Kg/day during 7 days, were more extensive and characterized by the absence of the OHC cilia, derangement in the W pattern of the cells and folded cilia. The morphological evaluation observed in the basal turn occurred only in the third turn of outer hair cells (OHC). In E3 was observed severe damaged in the 3 rounds of OHC (Figure 4).

In group 5 (100mg/kg/day of Ginkgo biloba and, after 90 minutes, methamidophos 3,0 mg/Kg/day during 7 days), all the outer hair cells kept its normal shape and arrangement at the end of the experiment, as displayed in Figure 5.

The apical turn was not analyzed in this study because it detects the low sound tones, while the ototoxic damages mainly detects the high tones. Besides, this turn has a natural disarrangement in its cells, leading to difficulty in the anatomical analysis. Furthermore, the CCI was not observed in this study because they are the least damaged cells when exposed to the ototoxicity, compared to the OHC.

## DISCUSSION

In Otology, the guinea pigs are largely used as experimental animals because of its easy handling, good cochlear dissection and manipulation as well as the facility to infuse anesthetic drugs and experimental drugs, intraperitoneally or intramuscularly^8^, ^11^, ^14^.

The most common type of OHC injury found on the analysis was the absence of sterocilia, followed by the cilia distortion with derangement of “W” pattern. This finding coincides with other publications^11^, ^15^.

Since the end of the ‘80s until the present date, studies have been made in order to comprehend the mechanisms of ototoxicity and the drugs that could be otoprotector for ciliated cells. We believe that the research field, which studies the endogenous self-defense mechanisms of hair cells of Corti's organ, in conjunction with genetic research and functional evaluation, is the most rational way to guide into concrete results that allows a better control over the injury and prevention of toxicity in the inner ear.

The action of pesticides on the nervous system causes late symptoms, which means, they do not occur immediately after the acute poisoning^7^, ^16^. At low and repetitive doses, the organophosphate possibly induces loss or reduction of neuronal branches^17^.

The results confirm the tendency for morphological injury progression according to the organophosphate intoxication level, since the largest number of injuries was observed in group 4 in comparison to group 2, reminding that the first guinea pigs group received a higher dose of pesticide.

Analyzing the findings from SEM, it is possible to infer that the injuries caused by organophosphate in the Corti's organ are predominantly in the OHC and progress from apex to the base of the cochlea. In the analyzed cochlea turns, the first OHC row is the first to be injured, followed by the second and third rows. This sequence of injuries coincides with the OHC height, being E3 the first to be injured; the second turn that suffers greater damage due to the organophosphate is E2 and the basal turn is the third one with many alterations.

Previous studies on guinea pigs submitted to ototoxic drugs diverge from the present study on the SEM observation. When the guinea pigs were exposed to amikacin antibiotic, the injuries predominated in the first two turns, and presented lower normal OHC in comparison to E3, which structural alterations confirm the study^18^ that found more intense changes in the first row of OHC, followed by the second row. Similarly, another study^13^ showed that aminoglycosides damage the Corti's organ mainly on OHC, with progression of aggression from the base to the apex of the cochlea, being the first row of OHC the first to be injured, followed by to the second and third rows.

In researches where cisplatin was used, the authors^19^ described extensive injuries on OHC of basal turn of the cochlea, in agreement to another study^15^, where the authors found more evident alterations on basal turn, with absence of cilia in the three rows of OHC, followed by E2 and E3, respectively. There were cilia changes in the inner ciliar cells, with cilia present, but disordered.

In this study, the animals who received the Ginkgo biloba extract before the pesticide, maintained the OHC cytoarchitecture and showed no structural changes in the Corti's organ, in agreement with study on rats^9^. Those studies demonstrated the otoprotective effect of Ginkgo biloba to OHC exposed to cisplatin. 90 minutes after the animals receive 100 mg/kg/day of Ginkgo biloba, it was used a dose of 1.0 mg/kg/day for 10 consecutive days of cisplatin, and after that, the measure of the action potential by electron microscopy demonstrated the protective effect of Ginkgo biloba.

The EGb 761 acts as a cleaner of free radicals, because it prevents the lipid-peroxidation, increases levels of GSH and the activity of antioxidant enzymes, and also has a superoxide dismutase activity (SOD)^20^. As a result, EGb 761 contributed to prevent the methamidophos' ototoxic effect. Its active principles, in particular, ginkgolides and flavonoids, are responsible for the antioxidant effects, and they supposedly migrate to the inner ear^9^, so this study showed that EGb 761 had effects on the toxicity induced by organophosphate - methamidophos.

The Ginkgo biloba has antioxidant properties and acts by decreasing lipid-peroxidation, increasing levels of catalase, superoxide dismutase and glutathione and removing the intracellular superoxide anions and free radicals. In plasma, it also acts forming complexes with phospholipids, increasing its antioxidant activity. In intracellular level, it acts on oxidative phosphorylation of mitochondrial RNA and on Golgi apparatus, supporting the mechanism of DNA repair and improving the “antioxidant status”, with increase on intracellular glutathione levels and decrease on 3H -timidina^21^ incorporation.

In agreement with other authors^10^, it was observed that there is a protective effect of Ginkgo biloba on the outer hair cells, since we found no damage on outer ciliar cells in SEM, or on the basal turn of the cochlea, or on E2 and E3 turns, after 7 consecutive days of pesticide used on doses of 0.3 mg/kg/day and 3.0 mg/kg/day, when used in combination with Ginkgo biloba.

Considering the ototoxicity mechanisms related to cell antioxidation and free radical formation, as well as being the Ginkgo biloba extract favorable to lipid-peroxidation lowering, with superoxide cleaning and prevention of free radicals formation, it should be considered the possibility of using Ginkgo biloba on the exposure to methamidophos, aiming to minimize its ototoxic effects. Besides the extract of Ginkgo biloba is being widely used in humans and have side effects considered being negligible, it also showed evidence of otoprotection to methamidophos, related to the OHC on the cochlea of guinea pigs exposed to organophosphate.

## CONCLUSION

According to what was observed utilizing SEM, it is possible to conclude that, the longer the exposition to the pesticide, bigger would be the outer hair cells damage in the guinea pigs. Instead, the guinea pigs who had previously received the Ginkgo biloba had their outer hair cells in the inner ear morphologically intact.
